# Rare and Unusual Consequences of Blunt Abdominal Trauma—The Significance of Anatomical Anomalies

**DOI:** 10.3390/jcm15082842

**Published:** 2026-04-09

**Authors:** Maciej Rybicki, Bartłomiej Białas, Wiktoria Jachymczak, Igor Karolczak, Julia Kot, Klaudia Dobrowolska, Bartosz Marek Czyżewski, Joanna Czyżewska, Kamil Paszowski, Karol Kamil Kłosińki

**Affiliations:** 1 Student’s Research Group, Department of Biomedicine and Experimental Surgery, Faculty of Medicine, Medical University of Lodz, Narutowicza 60, 90-136 Lodz, Poland; bartlomiej.bialas@stud.umed.lodz.pl (B.B.); wiktoria.jachymczak@stud.umed.lodz.pl (W.J.); igor.karolczak@stud.umed.lodz.pl (I.K.); julia.kot@stud.umed.lodz.pl (J.K.); klaudia.dobrowolska@stud.umed.lodz.pl (K.D.); bartosz.czyzewski@stud.umed.lodz.pl (B.M.C.); joanna.nadaj@stud.umed.lodz.pl (J.C.); 2 Plastic, Reconstructive and Aesthetic Surgery Clinic, Institute of Surgery, Medical University of Lodz, 90-151 Lodz, Poland; 3 Department of Biomedicine and Experimental Surgery, Faculty of Medicine, Medical University of Lodz, Narutowicza 60, 90-136 Lodz, Poland; kamil.paszowski@umed.lodz.pl

**Keywords:** blunt abdominal trauma, anatomical anomalies, visceral injury, diagnostic challenges, surgical management, computed tomography

## Abstract

**Background/Objectives**: Blunt abdominal trauma is a frequent challenge in emergency medicine, but its diagnosis and treatment become significantly more complex when rare anatomical anomalies are present. Atypical anatomy may mask symptoms or mimic other acute abdominal conditions, causing delays in treatment. The aim of this paper is to review the literature on six rare anatomical anomalies and their impact on the consequences of blunt abdominal trauma. **Methods**: A Narrative literature review was undertaken, covering PubMed, Scopus, Web of Science and Google Scholar databases, analysing publications from 1960 to 2025. Case reports and case series (91 patients in total) with confirmed organ damage following blunt trauma in the course of: duodenal diverticulum, Meckel’s diverticulum, splenic torsion, rupture or torsion of the accessory spleen, visceral inversion (situs inversus) and horseshoe kidney. **Results**: Demographic analysis revealed a predominance of perforations of the duodenal diverticulum in older women (mean age 62 years), while younger men predominated in all other groups. The clinical picture was often non-specific or misleading, especially in situs inversus, where the location of pain did not correlate with the typical topography of organs. Contrast-enhanced computed tomography (CECT) has proved to be a key diagnostic tool, surpassing ultrasound/FAST scans due to its ability to provide precise anatomical imaging. Surgical treatment was predominant (100% in Meckel’s diverticulum, 95% in duodenal diverticulum), while conservative treatment was effective in horseshoe kidney injuries (94.8%). Mortality was highest in situs inversus (29%) and duodenal diverticulum perforation (20%). The vast majority of these fatal cases occurred in the era of modern computed tomography, suggesting that the therapeutic challenges stem directly from the specific nature of these anomalies, rather than from past diagnostic limitations. **Conclusions**: Anatomical anomalies significantly modulate the clinical manifestations of blunt abdominal trauma, increasing the risk of diagnostic errors. Early contrast-enhanced computed tomography and awareness of these rare pathologies are crucial for appropriate management and improved prognosis.

## 1. Introduction

Injuries constitute one of the most significant challenges in contemporary emergency medicine and trauma surgery, representing the leading cause of death in the first half of life, and in the general population, they constitute the fourth leading cause of mortality [1]. Abdominal injuries are especially common, particularly blunt traumatic injuries, which accompany nearly 15% of patients admitted to Accident and Emergency departments [2].

Among the circumstances in which blunt abdominal injuries are sustained, road traffic accidents are the most significant, accounting for approximately 40% of post-traumatic cases. The other equally numerous subgroups are patients with injuries suffered as a result of railway accidents and falls from height (each approximately 20% of cases). Although less frequently encountered, injuries resulting from direct physical aggression constitute a smaller proportion of cases and are often characterized by more localized patterns of injury [3,4,5,6].

In abdominal injuries following blunt trauma, parenchymal organs are most affected, including the liver (up to 38% of cases), spleen (33%) and kidneys (23%) [5]. According to some sources, over half of all injuries following blunt abdominal trauma involve the spleen and liver [5].

Due to the frequency of injuries in these areas, physicians expect them and actively screen for them. The picture is different for less common injuries associated with numerous and not-so-rare anatomical abnormalities. It should be emphasized that anatomical abnormalities do not usually interfere with the proper functioning of a given organ, so they may go unnoticed, as they do not generally cause pathological symptoms [7,8].

Presence of anatomical anomalies may alter the distribution of forces exerted on the organs, leading to unusual—often less obvious—mechanisms of injury. Furthermore, anatomical abnormalities may complicate diagnosis, delay treatment and potentially result in a poorer prognosis for patients with abnormalities compared to anatomically average patients with typical anatomical configurations [9].

Moreover, these variations may mask the clinical picture and hinder the interpretation of imaging tests, which directly affects the patient’s prognosis. which directly affects the patient’s prognosis. A classic example is the horseshoe kidney, which, due to the peripheral location of the isthmus and the lack of protection by the rib arches, is more vulnerable to mechanical injury [10,11,12]. A similar diagnostic challenge may be posed by Situs inversus. It is estimated that in over 80% of patients with this condition, the congenital abnormality was previously undiagnosed. Such a situation may contribute to misdiagnosis and may lead to delayed treatment or therapeutic mistakes [13]. The present narrative review summarises the available knowledge and compares the published data on abdominal traumatic injuries with rare anatomical abnormalities, which include duodenal diverticulum perforation, Meckel’s diverticulum injury, splenic pedicle torsion, rupture of the accessory spleen, Situs inversus, and horseshoe kidney injury.

The small number of publications and the lack of well-structured and comprehensive data, especially in comparative analyses, constitute a major obstacle to understanding the mechanisms and clinical implications of rare abdominal injuries with accompanying anatomical abnormalities. Although these injuries are extremely rare, they can be associated with a high risk of serious clinical consequences, even leading to death. This research gap was the direct reason for the development of this study.

Our intention is to create a practical and educational publication that will encourage all doctors, especially clinicians who directly treat trauma patients, to deepen their knowledge in this demanding field. The subject matter is presented in a concise, comparative form, constituting a critical synthesis of research findings and descriptions of specialist case studies collected in the literature on the subject over the last seven to eight decades.

## 2. Materials and Methods

### 2.1. Search Strategy

Comprehensive literature searches were conducted to identify publications describing cases of blunt abdominal trauma in patients with specific anatomical abnormalities. The review covered the major databases: PubMed, Scopus, Web of Science, and Google Scholar. All professional literature from 1960 to 2025 was screened, with the last update of the search performed in December 2025.

The search strategy was based on two mandatory conceptual blocks:

(1) terms related to trauma and (2) terms related to anatomical variations. In order for a publication to be eligible, it had to contain at least one term from each of the above-mentioned blocks. Boolean operators (AND, OR) and a combination of MeSH terms and keywords in the form of free text keywords were used.

Trauma block:

‘blunt abdominal trauma’, ‘blunt abdominal injury’, ‘parenchymal organ injury’.

Abnormalities block:

‘duodenal diverticulum’, ‘Meckel’s diverticulum’, ‘situs inversus’, ‘horseshoe kidney’, ‘splenic torsion’, ‘wandering spleen’, ‘accessory spleen’.

No formal systematic review protocol was applied due to the narrative nature of the study.

### 2.2. Eligibility and Exclusion Criteria

To ensure the reliability and accuracy of the analyzed data, clearly defined eligibility criteria were applied.

Inclusion criteria:

- Publications covering patients regardless of age,
- Confirmed cases of blunt abdominal trauma leading to tissue damage (e.g., perforation, rupture, haemorrhage, ischaemia),
- Pre-existing anatomical anomalies mentioned in the search strategy.
- Confirmation of abnormalities in imaging studies or during surgical intervention,
- Only case reports, case series, and original scientific articles were included.

Exclusion criteria:

- Injuries of a different aetiology than blunt (e.g., penetrating, iatrogenic) or of unknown origin
- Anatomical anomalies detected incidentally, without traumatic components.
- Animal testing
- Grey literature, including conference abstracts and unpublished manuscripts.

The applied literature search strategy and eligibility criteria are summarised in Table 1.

### 2.3. Selection and Verification Process

Selection was a multi-stage process and included:

1. Titles were analysed with the aim of rejecting publications that were clearly unrelated to the subject matter,
2. Abstracts were analysed to identify works that potentially met the inclusion criteria,
3. Full-text analysis of articles selected in earlier stages.

Each case was independently verified by at least two authors in terms of diagnosis, injury mechanism and treatment outcomes. Any discrepancies were resolved through discussion and consensus.

### 2.4. Data Collection Process

Each included article provided a standardised range of clinical data, including:

- Demographic data: age, gender,
- Characteristics of the injury: mechanism and circumstances of the event (e.g., traffic accident, fall from height),
- Clinical presentation: predominant symptoms, vital signs,
- Diagnostic imaging: methods used (CT, ultrasound, X-ray) and key findings,
- Therapeutic procedure: surgical or conservative treatment, type of procedures performed,
- Treatment outcomes: length of hospital stay, complications, mortality.

Due to the extremely rare and highly heterogeneous nature of the cases analysed, no meta-analysis or advanced statistical analyses were performed. The analysis was restricted to basic descriptive statistics, such as minimum, maximum and mean values.

### 2.5. Study Design

This paper is a narrative review, which is a conscious choice resulting from the exceptional rarity of the analysed conditions and the lack of comparative or prospective studies. The aim of the review is to:

- synthesise available case descriptions,
- identify common clinical patterns,
- summarise rare and diagnostically difficult consequences of blunt abdominal trauma,
- facilitate clinicians in recognising these atypical conditions,
- popularise knowledge in an accessible and structured form.

The purpose of this review is to organise scattered knowledge and identify common elements that may be of practical importance in the daily work of emergency physicians, surgeons and radiologists.

## 3. Results

### 3.1. Traumatic Perforation of Duodenal Diverticula

#### 3.1.1. Clinical Background of Traumatic Perforation of Duodenal Diverticula

Diverticular perforation of the duodenum is a rare but not insignificant consequence of blunt abdominal trauma. To date, approximately 200 cases of diverticular perforation of the duodenum have been reported in professional literature [14], of which less than 25 cases involved blunt abdominal trauma [15,16,17,18,19,20,21,22,23,24,25,26,27,28,29,30,31,32,33,34,35,36,37].

It should be emphasised that, despite the prevalence of duodenal diverticula in the population—autopsies reveal them in up to 20% of people—up to 95% of them remain asymptomatic throughout life [38,39,40]. The majority of cases (62%) are located in the vicinity of Vater’s papilla [41]. Only a small proportion are located in the horizontal (D3) part of the duodenum. Duodenal diverticula are most often divided into congenital and acquired. The congenital form accounts for only 10% of all cases, develops during prenatal life and has a full wall, just like the duodenum. Acquired diverticula, on the other hand, account for as much as 90% of cases, develop during the course of life and have a thin wall composed of mucosa and serosa [42].

#### 3.1.2. Patient Characteristics with Traumatic Perforation of Duodenal Diverticula

Typically, patients who suffer from diverticular perforation following blunt trauma are 62 years old on average (±19 years). Women slightly outnumber men in this group (57% of cases) [43]. The majority of injuries are caused by traffic accidents (43%) and falls from height (38%).

#### 3.1.3. Traumatic Mechanisms Associated with Perforation of Duodenal Diverticula

In terms of the mechanism of duodenal diverticulum perforation, we propose the following aspects: compression of the diverticulum between an external force and the vertebral column, a sudden increase in intraintestinal pressure, and shear forces during deceleration. The diversity of mechanisms (traffic accidents vs. falls vs. local injuries such as kicks or blows) further suggests that the mechanism of injury may influence the location and course of the rupture [43].

#### 3.1.4. Locations of Traumatic Perforation of Duodenal Diverticula

Duodenal diverticula are most commonly located in the descending part (D2), on the medial wall, near the bile and pancreatic ducts. This location may promote outflow disorders and infections, which may explain the higher incidence of perforations in the D2–D3 segments [43,44].

#### 3.1.5. Clinical Manifestation of Traumatic Perforation of Duodenal Diverticula

The symptoms of traumatic perforation of the duodenal diverticulum are non-specific and may resemble other, much more common conditions, including acute cholecystitis, gastric and/or duodenal ulcer disease, acute pancreatitis, and acute appendicitis [41,45,46].

The most common symptoms accompanying duodenal diverticulum perforation after blunt trauma include abdominal pain (81%), epigastric tenderness (71%), and, less frequently, nausea or vomiting (24%) or peritoneal symptoms (19%) [43]. Such clinical manifestations may delay and complicate accurate diagnosis, and thus may lead to delayed treatment, reducing the patient’s chances of recovery.

#### 3.1.6. Diagnosis of Traumatic Perforation of Duodenal Diverticula

With the development of imaging techniques, including endoscopy, duodenal diverticula are increasingly being detected incidentally during routine ERCP/gastroscopy examinations. However, it should be mentioned that computed tomography (CT) has significantly improved the detection rate of duodenal diverticula, especially the most common type—peri-papillary diverticula. To date, CT remains the gold standard in the diagnosis of both duodenal diverticulum perforation and other parts of the gastrointestinal tract [47].

Noteworthy is the fact that typical radiological symptoms of perforated diverticula are similar to those occurring in perforations of other parts of the digestive tract. These include: the presence of extraluminal air, inflammatory infiltration in the retroperitoneal space, fluid collections, and contrast medium leakage outside the duodenal lumen [14,48,49].

Additionally, other imaging methods such as ultrasonography (detected 3/5 cases) and X-ray (detected 2/6 cases) may be helpful. However, it should be emphasised that among the cases described, only CT detected perforation in each case, while the effectiveness of ultrasound reaches 60% and X-ray 33%.

#### 3.1.7. Treatment of Traumatic Perforation of Duodenal Diverticula

Almost all cases (20/21) of traumatic perforation of a duodenal diverticulum involving the gastrointestinal tract are treated surgically, which is the gold standard of treatment.

The most common surgical methods of treatment include manual diverticulectomy (wedge resection of the diverticulum) and stapler resection with continuity restoration. It is interesting that in almost all cases of surgical treatment, retroperitoneal drainage was used (18/20 surgical cases). Interestingly, the literature describes a case of diverticular perforation after blunt abdominal trauma, which was successfully treated conservatively [35]. In the available case reports, treatment was most often surgical, although in non-traumatic situations, conservative management (antibiotic therapy, monitoring) or endoscopic treatment in cases of limited perforation was reported to be effective [50,51]. Furthermore, the length of hospitalisation can significantly vary from 4 days [32] to even 2 months [20].

#### 3.1.8. Analysis of Results

Post-traumatic perforations of duodenal diverticula, despite their extreme rarity, pose a significant diagnostic and therapeutic challenge. According to available data, mortality in this group of cases reaches approximately 20% [43]. Due to the non-specific clinical picture, duodenal diverticulum injury should be considered in the differential diagnosis of the so-called quiet abdomen after trauma [52,53]. The literature data are limited—most publications include single case reports, two case series [34] and one systematic review of the literature [43]. Contrast-enhanced computed tomography remains the diagnostic method of choice for the diagnosis of gastrointestinal perforation, including duodenal diverticular perforation [54,55].

#### 3.1.9. Summary of Traumatic Perforation of Duodenal Diverticula

Duodenal diverticulum perforation following blunt abdominal trauma is a rare occurrence [15,16,17,18,19,20,21,22,23,24,25,26,27,28,29,30,31,32,33,34,35,36,37], mainly affecting women with a mean age of 62 years [43]. The mechanism of injury usually involves compression or a sudden increase in intraintestinal pressure during traffic accidents, and the clinical symptoms are non-specific and may delay diagnosis [41,46]. The gold standard for detecting perforations remains computed tomography, which is significantly more effective than ultrasounds or X-rays [47]. The treatment of choice is surgical intervention, which usually involves excision of the diverticulum with retroperitoneal drainage, although the literature also describes a case of successful conservative treatment [35]. The mortality rate in this group of patients is around 20%, which emphasizes the need to include this injury in the differential diagnosis [43,52].

### 3.2. Traumatic Perforation of Meckel’s Diverticulum

#### 3.2.1. Clinical Background of Blunt Injuries of Meckel’s Diverticulum

Meckel’s diverticulum (MD) is the most common congenital anomaly of the gastrointestinal tract, representing a true diverticulum of the ileum that may contain heterotopic tissue, most frequently gastric or pancreatic mucosa, though carcinoid, duodenal tissue, lipoma, mucocele, leiomyoma, metastatic adenocarcinoma, jejunal mucosa, and Brunner’s glands have also been described. While most MDs are asymptomatic and discovered incidentally, a wide range of complications has been documented, including obstruction, inflammation, haemorrhage, perforation, neoplastic transformation, and inclusion within a hernial sac, with traumatic perforation being an exceptionally rare complication [56,57,58,59,60].

#### 3.2.2. Patient Characteristics with Blunt Injuries of Meckel’s Diverticulum

Blunt traumatic injury to the MD is exceedingly rare. Up to December 2025, a total of 30 complete case reports have been published [61,62,63,64,65,66,67,68,69,70,71,72,73,74,75,76,77,78,79,80,81,82,83,84,85,86,87,88]. In the study by Arkuszewski et al. (2024) [89], analysing 28 of these cases, the vast majority of patients were male (96.4%), with a mean age of 27.3 years. The majority of traumatic MD injuries resulted from traffic accidents, followed by sports-related and occupational incidents, and, less frequently, acts of violence. In some cases, the diverticular injury was accompanied by trauma to other intra-abdominal organs, most commonly the spleen or liver, necessitating surgical intervention [89]. The marked male predominance may be explained by greater exposure of young men to high-energy trauma, particularly road traffic accidents and high-impact occupational or sports activities, as well as age-related risk-taking behaviours, which together increase the likelihood of blunt abdominal injury affecting Meckel’s diverticulum.

#### 3.2.3. Traumatic Mechanisms Associated with Blunt Injuries of Meckel’s Diverticulum

Proposed mechanisms of post-traumatic MD perforation include:

- Pressure rupture from rapid transmission of elevated intra-abdominal pressure to the diverticular lumen or solely from increased intra-abdominal pressure [62,63,67,80,81,85];
- Crushing injury between the abdominal wall and rigid structures such as the vertebral column? [85,90];
- Shearing forces during rapid deceleration [63,67,81];
- Possible contribution from pre-existing inflammation or ectopic mucosa, which may weaken the diverticular wall [62,76,88].

In cases of post-traumatic intussusception involving MD, the proposed mechanism involves altered peristalsis and segmental dysmotility after abdominal injury, with MD acting as a fixed lead point around which the proximal bowel telescopes [66].

#### 3.2.4. Locations of Blunt Injuries of Meckel’s Diverticulum

Perforation of the MD may occur at the tip [63,64,67,68,72,80,84,91], base [67,85,88,92], distal segment [62,81,87], the central portion of the diverticulum [61].

#### 3.2.5. Clinical Manifestations of Blunt Injuries of Meckel’s Diverticulum

Based on the study by Arkuszewski et al., traumatic involvement of MD most commonly manifests as isolated perforation, followed by bleeding from the diverticulum, perforation associated with mesenteric tearing and haemorrhage, and, less frequently, intussusception of the diverticulum or intramural haematoma with an adjacent mesenteric haematoma [89]. MD may also form part of the contents of a traumatic abdominal wall hernia [83].

Clinically, these injuries present with a highly variable and often nonspecific spectrum of symptoms, most often resembling an acute abdomen. Patients typically report abdominal pain, distension, vomiting, and tenderness, with frequent findings of intestinal obstruction, peritoneal irritation, or generalised peritonitis. In more severe cases, hollow viscus perforation, intra-abdominal bleeding, and haemorrhagic shock may occur. Radiological evaluation commonly reveals free intraperitoneal fluid, pneumoperitoneum, dilated small-bowel loops, or hemoperitoneum, reflecting the wide range of severity of these traumatic presentations.

Compared with traumatic perforation of a duodenal diverticulum, which more commonly presents with isolated epigastric pain with potentially delayed peritoneal symptoms, traumatic perforation of MD tends to produce more evident features of acute abdomen, frequently with peritoneal signs, which may facilitate earlier clinical suspicion.

#### 3.2.6. Diagnosis of Blunt Injuries of Meckel’s Diverticulum

In none of the reported cases was traumatic MD diagnosed preoperatively; however, in all instances, the underlying condition necessitating surgical intervention was correctly identified. Preoperative assessment primarily relied on clinical evaluation, which frequently revealed signs of intestinal obstruction (abdominal distension, colicky pain, vomiting), peritoneal irritation, or features of haemorrhagic shock. Imaging studies, including plain abdominal radiographs, ultrasonography (USG), and computed tomography (CT), were particularly useful in identifying indirect signs of injury, such as pneumoperitoneum, free intraperitoneal fluid, dilated small-bowel loops, intussusception, or intra-abdominal bleeding. Despite these diagnostic tools, the definitive recognition of MD injury was possible only intraoperatively, where perforation, haematoma, or associated mesenteric damage was confirmed. In contrast to duodenal diverticulum (DD), in which CT imaging can often directly demonstrate perforation, in the case of MD, CT typically detects the resulting complication rather than identifying the diverticulum itself as the primary source of pathology. This highlights the limited specificity of preoperative imaging in detecting MD as the direct source of injury.

#### 3.2.7. Treatment of Blunt Injuries of Meckel’s Diverticulum

In all cases, the diagnosis of MD injury was confirmed intraoperatively. Treatment was tailored to the lesion type and severity, including wedge resection of the MD with primary bowel closure for small perforations, or segmental ileal resection with end-to-end or side-to-side anastomosis for broad-based diverticula, extensive perforation, ischemia, or mesenteric injury. Haemostasis was achieved by ligating bleeding mesodiverticular vessels or mesenteric branches. Intussusception was reduced manually or treated with bowel resection in cases of ischemia. Additional procedures, such as appendectomy or mesenteric defect closure, were performed when indicated. Surgical management aimed to remove the injured diverticulum, restore bowel continuity, control bleeding, and prevent further peritoneal contamination.

#### 3.2.8. Analysis of Results

Blunt traumatic involvement of the MD is extremely rare, occurring mainly in young male patients after high-energy trauma. Clinical presentation is nonspecific, and preoperative diagnosis is uncommon, with MD involvement typically identified intraoperatively. While diverticular perforation is the most frequent manifestation, other forms such as intussusception, mesodiverticular bleeding, intramural haematoma, or traumatic abdominal wall herniation containing MD also contribute to morbidity. Surgical management, tailored to intraoperative findings, remains the treatment of choice and is associated with favourable outcomes when performed promptly.

#### 3.2.9. Summary of Blunt Injuries of Meckel’s Diverticulum

Traumatic perforation of the MD is a rare but potentially life-threatening condition. Awareness of various manifestations, combined with timely surgical intervention, is essential for preventing complications and ensuring favourable patient outcomes.

### 3.3. Splenic Torsion

#### 3.3.1. Clinical Background of Splenic Torsion

Splenic torsion is a rare occurrence, with approximately 500 cases described worldwide [93].

The condition is mostly associated with spleen hypermobility (wandering spleen). In adults, abnormal spleen mobility is considered to be caused by trauma, multiparity, previous splenic surgery, or splenomegaly. In children, hypermobility is caused by congenitally defective development of the splenic ligaments [94].

The spleen is anchored by four ligaments: splenorenal, splenocolic, gastrosplenic, and splenophrenic [95,96].

In this condition, the spleen twists along its vascular pedicle [97].

With the splenic vessels running within the pedicle, its torsion can lead to partial or complete infarction of the spleen. Although splenic torsion is typically associated with wandering spleen, blunt abdominal trauma may precipitate torsion in patients with predisposing ligamentous laxity [98].

#### 3.3.2. Patient Characteristics with Splenic Torsion

Splenic torsion as a result of blunt abdominal trauma is a limited number of reported cases [99,100,101,102,103,104,105,106], which suggests that this complication is a rare occurrence.

The patient’s group consisted of 8 people. Their age was within the 5–28 range. The median age of the group was 15.5 years, and 62.5% of the patients were below the age of 18. The group consisted of an equal number of male and female patients.

#### 3.3.3. Traumatic Mechanisms Associated with Splenic Torsion

In two cases, the nature of trauma was described as blunt and abdominal. Five cases were associated with traffic accidents. Two case reports do not include clear descriptions of the trauma. It is worth mentioning that the most common cause of blunt abdominal trauma is motor vehicle accidents [107].

In post-traumatic splenic torsion, the force of trauma causes twisting of the splenic pedicle, thus reducing blood supply to the organ and causing ischemic damage. In the majority of cases, the spleen was twisted along the long axis of its pedicle. In all of the cases, a wandering spleen was found during the operation. However, in one case, an anomalous spleen localization was discovered prior to trauma [99]. The abnormal position reflects the absence or laxity of supporting splenic ligaments. These findings suggest that blunt trauma acts as a triggering factor in patients with pre-existing splenic hypermobility.

#### 3.3.4. Locations of Splenic Torsion

In the majority of cases, the spleen was twisted along the long axis of its pedicle. An abdominal mass may be palpable in varying abdominal regions, ranging from the middle abdomen to the lower pelvis. The abnormal position reflects the absence or laxity of supporting splenic ligaments.

#### 3.3.5. Clinical Manifestation of Splenic Torsion

Clinical signs and symptoms of post-traumatic splenic torsion include abdominal pain that varies in location, a tender abdomen, fever, nausea, vomiting, peritoneal signs and leukocytosis. An abdominal mass may be palpable in varying abdominal regions, ranging from the middle abdomen to the lower pelvis.

#### 3.3.6. Diagnostic of Splenic Torsion

A definitive diagnosis of splenic torsion can be made with both enhanced computed tomography and colour Doppler ultrasonography. Radiological findings include the absence of spleen in its physiological location and an ovoid mass in a different location, with fluid in the abdomen. The use of contrast-enhanced computed tomography shows that the mass does not enhance or the enhancement is minimal, which suggests impaired blood supply. The splenic vessels may demonstrate a “whirl” or corkscrew-like appearance and may appear widened and non-enhancing, suggesting torsion of the splenic pedicle.

#### 3.3.7. Treatment of Splenic Torsion

Treatment of splenic torsion is related to the duration of the symptoms and the severity of splenic infarction. Splenic preservation in the form of splenopexy should be a priority, and if it is not possible, splenectomy should be performed [97].

In the majority of cases (87.5%), the treatment of choice was splenectomy. However, post-traumatic splenic torsion may not always involve spleen resection. Only in one case was it possible to perform a successful splenopexy of a torsioned spleen. The treatment took place 2 months after the trauma [102]. This information is crucial as splenectomy carries an increased risk of portal vein thrombosis, overwhelming post-splenectomy infection (OPSI) and a lifelong increased risk of severe infections [108].

In six out of eight cases, the operation method was laparotomy. In the remaining two, the method was not described.

#### 3.3.8. Analysis of Results

The period from trauma to surgery varies, with the shortest one being 3 days and the longest 2 months [102].

In one case [101], the lack of correct splenic torsion diagnosis led to splenic avulsion and infarction. However, the splenic capsule remained intact, which further supports the notion that the spleen avulsion was secondary to torsion and that it was not a primary traumatic avulsion.

In another case, trauma was considered to cause spleen hypermobility, which was followed by spontaneous torsion after 8 weeks [105]. These findings suggest that trauma may either directly induce torsion or act as a precipitating factor in patients with pre-existing splenic ligamentous laxity.

#### 3.3.9. Summary of Splenic Torsion

In conclusion, the described cases depict the clinical presentation of post-traumatic splenic torsion. The spleen is damaged by ischemia, not the energy of the trauma, and the damage does not have a primary structural-disruptive character. Perhaps, regarding the predisposition to splenic torsion, this pathology occurs in cases with splenic fixation disorders, and patients with proper spleen fixation have a smaller risk of post-traumatic splenic torsion. Available published cases suggest that post-traumatic torsion occurs mainly in patients with predisposing fixation abnormalities, but the evidence is insufficient to draw definitive conclusions. This suggests that post-traumatic splenic torsions only affect wandering spleens and that blunt trauma would not have the same effect on a properly fixed spleen. However, verification of this hypothesis requires further research on a larger group.

### 3.4. Rupture and Torsion of Accessory Spleen

#### 3.4.1. Clinical Background of Accessory Spleen

The accessory spleen (AS) is a congenital anomaly, which is defined by a separate cluster of splenic tissue that arises apart from the primary spleen [109,110]. The incidence of AS in autopsy studies is estimated to be 10–40%; moreover, AS is found in 45–65% of patients who have undergone splenectomy [111] primary structural-disruptive character [112]. The two major consequences of trauma concerning AS are rupture and torsion, although both of these conditions are extremely uncommon [113].

#### 3.4.2. Patient Characteristics with Assessory Spleen Injuries

- Rupture of AS

Data on the characteristics of individuals with rupture of AS is insufficient. In an analysis from 2024, in 8 of the 9 reported cases, the patients were male (89%), and the mean age of all patients was 35 years; nevertheless, patients ranged in age from 8 to 61 years [113].

- Torsion of AS

The majority of cases of AS torsion occur in paediatric patients; however, patients range in age from infants to the elderly [114]. In the single documented case of traumatic torsion of AS, the patient was a 12-year-old male [115].

#### 3.4.3. Traumatic Mechanisms Associated with Rupture and Torsion of Assessory Spleen

- Rupture of AS

Rupture of the AS most commonly occurs as a consequence of direct trauma and typically is associated with blunt abdominal trauma [116]. The magnitude of trauma energy does not directly correlate with the risk of AS rupture, as some patients had the primary spleen removed due to trauma, and no injury to the AS occurred [113].

- Torsion of AS

In the literature, there is only one reported case of post-traumatic torsion of AS, therefore, the data on the mechanism causing this type of injury remains limited. This condition results from twisting of the vascular pedicle of AS, which subsequently leads to infarction [115]. These findings suggest that anatomical factors, rather than trauma severity alone, may determine the risk of AS rupture.

#### 3.4.4. Locations of Assessory Spleen

AS most often occur in the splenic hilum (75%) and at the pancreatic tail (20–25%), but they may also be found in other abdominal regions [115,117].

#### 3.4.5. Clinical Manifestation of Assessory Spleen Torsion and Ruptures

- Rupture of AS

Acute abdominal pain is the predominant symptom of AS rupture, though progressive pain may be observed in some cases [116].

- Torsion of AS

Symptoms of AS torsion include acute abdominal pain, leucocytosis, fever, nausea and vomiting; however, pain localization varies according to the AS position [114].

#### 3.4.6. Diagnostic of Assessory Spleen Torsion and Ruptures

Contrast-enhanced computed tomography is the imaging method of choice for the diagnosis of both conditions, with ultrasound and X-ray as supportive tools [114,116].

- Rupture of AS

It is extremely challenging to identify post-traumatic rupture of AS, and in the analysis of nine case reports, the diagnosis was confirmed only in two cases based on the radiology assessment, whereas for the other cases, the diagnosis was determined during surgery [113]. Detection of ruptured AS on ultrasound and even CT (computed tomography) scan may be difficult because of its small dimensions and adjacent hemoperitoneum [116].

- Torsion of AS

Contrast-enhanced computed tomography is a useful imaging method for establishing the diagnosis of AS torsion; nevertheless, in most cases, the diagnosis is confirmed intraoperatively [114]. Small size and atypical location of AS significantly reduce the sensitivity of imaging studies.

#### 3.4.7. Treatment of Injuries Associated with Assessory Spleen

- Rupture of AS

The treatment of ruptured AS varies according to the patient’s hemodynamic stability. Based on cases described in the literature, it can be inferred that conservative management, which consists of fluid maintenance, bed rest and clinical observation, is recommended for patients who are hemodynamically stable. Laparotomy is required in cases of hemodynamic instability and suspicion of active bleeding [113,116]. A retrospective evaluation of case reports from 2024 indicated that traumatic rupture of AS predominantly required laparotomy, as conservative treatment was implemented in merely two of nine analysed cases [113].

- Torsion of AS

Laparoscopic splenectomy is the gold standard when it comes to the management of this condition. Single-port and single-port plus one laparoscopic approaches provide sufficient access. Although surgical intervention is traditionally considered necessary due to the risk of infarction, recent reports suggest that conservative management may be appropriate in selected cases- especially among paediatric patients with non-severe clinical course [118,119]. Non-surgical treatment can be considered if symptoms and blood tests are mild and there are no additional complications or signs of malignancy. Nevertheless, conservative management requires strict observation with contrast studies assessing the risk of re-torsion [120].

#### 3.4.8. Atypical Cases

A particularly favourable aspect of the presence of AS for patients with traumatic splenic rupture is the fact that AS may undergo compensatory hypertrophy after surgical removal of the primary spleen. However, the case study of a 45-year-old woman, who underwent removal of the primary spleen due to post-accidental rupture, demonstrates that the spontaneous rupture of hypertrophic AS, though uncommon, is possible [113,121].

Even though post-traumatic AS rupture is typically reported in younger patients, the case of a 61-year-old male illustrates that it may occur in individuals of any age. This patient experienced mild pain elicited on palpation of the left lower abdominal region and hematuria after participating in a motor vehicle collision. Interestingly, the patient had a history of splenectomy also due to a motor vehicle collision. CT scan revealed laceration of the accessory spleen, liver and right kidney, as well as left adrenal haematoma and hemoperitoneum. The patient was treated conservatively with a good outcome [122].

Torsion of the AS directly associated with blunt trauma is exceedingly rare, with only a single case reported in the literature. A 12-year-old male was admitted to the hospital due to pain in the left abdominal region, secondary to blunt trauma to the area. Although the abdominal X-ray showed no abnormality, diagnostic evaluation was expanded. Radiologic evaluation, including CT scan and ultrasound, demonstrated an oval 4 cm mass, located in the upper left abdomen, in close proximity to the pancreatic tail, distinctly separated from the spleen. CT scan detected a hypodense tubular structure in the region superior to the mass. Thus, the diagnosis of twisting of the vascular pedicle of the AS was established. The patient underwent a laparoscopic resection twenty-five days after the injury. The AS was adherent to the omentum and colon and had undergone four full rotations around its axis. The postoperative pathological examination confirmed the diagnosis of AS torsion [115].

#### 3.4.9. Analysis of Results

Confirming the diagnosis of rupture and torsion of AS is challenging and not always possible based on imaging studies. Another factor complicating diagnosis is the fact that an accessory spleen can have different locations, and also, clinical manifestations may vary depending on the position of this organ. Since both conditions are extremely infrequent, access to relevant literature sources is limited and largely based on case reports, there is a need to establish diagnostic and therapeutic standards. The rarity of these entities precludes the development of evidence-based guidelines and necessitates individualised clinical decision-making.

#### 3.4.10. Summary of Assessory Spleen Injuries

Management of these two conditions involves surgical intervention- most cases of AS rupture necessitate laparotomy [113] and torsion of AS is also treated surgically with laparoscopic splenectomy as the method of choice; however, a conservative approach is possible in certain cases, particularly in children with mild symptoms and no definite signs of full torsion [118,119]. Prior confirmation and awareness of AS presence may support diagnosing rupture or torsion of AS. Trauma-induced AS rupture or torsion is not linked to the injury of the primary spleen. Therefore, AS injury should be considered independently in the differential diagnosis of post-traumatic abdominal pain.

### 3.5. Situs Inversus

#### 3.5.1. Clinical Background of Situs Inversus

Good knowledge of body anatomy is particularly important in emergency medicine. However, there are cases with a very rare abnormality, visceral inversion (situs inversus). In the definition, situs solitus is the normal position of the cardiac atria and viscera, while situs inversus is its mirror image [123]. The frequency of situs inversus is 1:10,000 and is more frequent in males: 1.5:1 [124]. Situs inversus can be differentiated into situs inversus with levocardia or situs inversus with dextrocardia. When situs cannot be determined, the patient is said to have situs ambiguous or heterotaxy. In these patients, the liver may be located in the midline, the spleen may be absent or have multiple splenic tissues, the morphology of the cardiac atria may be unclear, and the intestines may be malrotated [123,125].

Situs inversus is typically an autosomal recessive genetic condition, though it can also be X-linked. Patients with situs inversus totalis have a 5–10% prevalence of congenital heart disease. The condition may also be associated with primary ciliary dyskinesia, known as Kartagener syndrome, which may or may not involve bronchiectasis. Primary ciliary dyskinesia is a heterogeneous disease with impaired mucociliary transport leading to respiratory disorders, hearing impairment and male infertility function [126,127].

While many individuals with this condition remain asymptomatic throughout life, the reversed organ placement causes diagnostic difficulties in acute care. E.g., left-sided abdominal pain may indicate pathology typically associated with right-sided organs [128]. In the context of trauma, reversed anatomy may delay diagnosis or lead to incorrect initial clinical assumptions.

#### 3.5.2. Patient Characteristics with Situs Inversus

The review is based on seven selected cases of patients with situs inversus and blunt trauma published in the literature between 1987 and 2024 [123,127,128,129,130,131,132]. The patients described were relatively young, with a mean age of ±23 years, and the majority were men (71%). The most common cause of blunt trauma was traffic accidents (43%). The remaining causes were related to an accident at work, at home, a fall from a height, or, in one case, it was not precisely specified.

#### 3.5.3. Traumatic Mechanisms Associated with Situs Inversus

Blunt abdominal trauma can occur through various mechanisms, depending on the underlying cause. In the case of a wooden block springing from a press machine [128], the injury was caused by compression. This can cause crushing and increased pressure inside the intestine. In addition, in road accidents [123,129,130] or the case of falling from a height [131], internal displacement of organs occurs relative to the abdominal wall, which can cause organ ruptures or tearing of supporting structures such as ligaments or the mesentery [4]. The mechanism of trauma itself does not differ in patients with situs inversus; however, the mirrored anatomical configuration may alter the clinical interpretation of symptoms and imaging findings.

#### 3.5.4. Locations of Situs Inversus

In the analysed cases, the most common injury involved the spleen (in 5 of the 7 cases), although the liver and intestines were also damaged. It is worth noting that in two cases [127,128], appendiceal inflammation occurred, which was likely a complication of peritonitis.

#### 3.5.5. Clinical Manifestation of Situs Inversus

Blunt abdominal trauma has a variety of clinical manifestations. The most common accompanying symptoms were abdominal tenderness (71%) and abdominal pain (57%), less frequently vomiting (29%), and muscular guarding (29%). In one case, symptoms appeared immediately after the event, were absent for 7 days, and then reappeared [128]. In the case of the patient who slipped and hit a kitchen table, symptoms appeared after 4 days [132]. In one patient, typical gastrointestinal symptoms were not described because his condition was extremely severe [130].

The initial diagnosis was misleading due to the location of the symptoms—the pain on the right side was related to a spleen injury, while the pain on the left side was caused by a damaged liver. This mirror-image presentation significantly increases the risk of delayed or incorrect initial diagnosis in emergency settings.

#### 3.5.6. Diagnostic of Situs Inversus

In all analysed cases, rapid and precise imaging diagnostics were of critical importance. Examinations such as abdominal radiography, ultrasonography, computed tomography (CT), and contrast-enhanced computed tomography (CECT) enabled not only the identification of injuries but also the confirmation of situs inversus. Typical radiological findings included dextrocardia, reversed positioning of the liver and gallbladder, as well as right-sided splenic location.

Computed tomography is more accurate than ultrasound in diagnosing abdominal injuries, as it allows for a detailed assessment of the solid organs, retroperitoneal space, and vessels, as well as the detection of minor ruptures, bleeding, and intestinal injuries, which often remain invisible on ultrasound. However, ultrasound remains a valuable screening tool—it is rapid, available at the bedside, and radiation-free, making it particularly useful in hemodynamically unstable patients, primarily for detecting free fluid in the abdominal cavity [127].

#### 3.5.7. Treatment of Injuries Associated with Situs Inversus

Almost all cases (6 of 7) were treated surgically. One patient was only hospitalised and observed [123], while the others underwent laparotomy.

Depending on the severity of the injury, procedures included splenectomy, intestinal resection, hemihepatectomy, and, in three patients, preventive appendectomy [127,128,132].

Non-operative management is used for blunt abdominal trauma in hemodynamically stable patients after ruling out the need for urgent laparotomy. Continuous monitoring of the patient and their vital signs is essential if their condition worsens and surgery is necessary. Immediate surgical treatment is indicated in life-threatening situations when internal haemorrhage is suspected, which may result in hypovolemic shock [133].

#### 3.5.8. Analysis of Results

This report aims to highlight the diagnostic difficulties associated with blunt abdominal trauma in patients with situs inversus. The mirror-image arrangement of internal organs may result in atypical symptom localization, which in emergency settings increases the risk of misinterpretation of the clinical presentation. In the analysed cases, the location of pain did not correspond to typical anatomical patterns; right-sided abdominal pain was associated with splenic injury, whereas left-sided pain resulted from liver trauma. This mismatch may lead to delayed diagnosis and inappropriate treatment.

Delayed recognition of abdominal injuries in patients with situs inversus may result in serious clinical consequences, including progressive internal haemorrhage, development of peritonitis, and deterioration of the patient’s hemodynamic status. In some of the reviewed cases, clinical symptoms appeared with a delay of several days after the traumatic event, further complicating the diagnostic process.

Among the seven analysed patients, two deaths were reported [127,129], showing the potentially severe course of blunt abdominal trauma in individuals with situs inversus and the importance of rapid and accurate diagnosis. In most cases (6 of 7), surgical treatment was required, indicating both the severity of injuries and the limited possibilities of non-operative management. Only one patient was managed exclusively non-operatively, confirming that non-operative management should be considered only in carefully selected, hemodynamically stable patients, under close clinical monitoring.

This study has several important limitations. The most significant is the small number of available cases, resulting from the rare coexistence of situs inversus and blunt abdominal trauma, which limits the ability to draw robust and generalised conclusions regarding diagnostic and therapeutic strategies. Furthermore, the analysed data are derived from case reports published over many years, leading to variability in diagnostic methods and treatment approaches. The retrospective nature of the review also restricts direct comparison of treatment outcomes.

Despite these limitations, this study emphasises the importance of awareness of anatomical variants in trauma patients and highlights the role of early, comprehensive imaging diagnostics, particularly computed tomography, in reducing diagnostic errors and therapeutic delays. In contrast to structural anomalies that predispose to organ injury, situs inversus primarily increases diagnostic complexity rather than intrinsic injury susceptibility.

#### 3.5.9. Summary of Situs Inversus

Situs inversus significantly complicates the diagnosis of blunt abdominal trauma, as atypical organ positioning may result in misleading symptom localization and delayed recognition of potentially life-threatening injuries, particularly in emergency situations. Early implementation of comprehensive imaging diagnostics, especially computed tomography, is crucial for accurate injury assessment and confirmation of abnormal organ laterality. Therapeutic management should be individualised; non-operative treatment may be considered only in carefully selected, hemodynamically stable patients under close observation, whereas immediate surgical intervention is required in cases of hemodynamic instability or severe intra-abdominal injuries.

### 3.6. Horseshoe Kidney Injuries

#### 3.6.1. Clinical Background of Horseshoe Kidney Injuries

A horseshoe kidney is one of the most common renal anomalies [134] occurring in about 1:400 patients with a 2:1 male-to-female ratio. Even though it is mainly asymptomatic [135], it may increase the probability and severity of sustained abdominal injury, with often confusing and complex imaging results [136] due to its position and various blood supply anomalies [134]. As in most renal injuries, observation or conservative treatment is preferred, but cannot always be achieved because of the horseshoe kidney’s complex anatomy [135]. The low position of the fused kidneys and the presence of an anterior isthmus reduce the protective effect of the rib cage, making the organ more vulnerable to blunt trauma.

#### 3.6.2. Patient Characteristics with Horseshoe Kidney Injuries

Previously pathological kidneys are responsible for 7% of renal injuries sustained due to abdominal trauma [135]. Thus, studies involving damage to horseshoe kidneys as a result of abdominal trauma are limited. In this study, a total of 12 adult cases [135,137,138,139,140,141,142,143,144,145,146,147] were analysed. All patients were male, with a mean age of 38.25 (range 21–78, SD = 17.06). The main cause of trauma was a motor vehicle accident (50%). As in overall renal traumas, rapid deceleration was the main mechanism, crushing kidney structures or avulsing blood vessels resulting in active internal bleeding and/or retroperitoneal haematoma. [https://uroweb.org/guidelines/urological-trauma/chapter/urogenital-trauma-guidelines, accessed on 6 December 2025] There were four cases involving paediatric patients [12,148,149,150] in which two were males and two females with a mean age of 13.5 (range 10–16, SD = 2.29).

#### 3.6.3. Locations of Horseshoe Kidney Injuries

Horseshoe kidney is a congenital condition caused by renal fusion anomaly during foetal development, characterised by ectopia, malrotation and vascular changes [151,152]. It occurs when the lower pole isthmus connects two kidneys from opposite sides of the spine, creating a U-shape, with an incidence estimated at around 1:400 and male predominance 2:1 [135,153]. The location of a horseshoe kidney is often lower than that of physiologic kidneys, just above the aortic bifurcation; however, it may appear even in the true pelvis, whilst the isthmus usually lies on the level of the 4th to 5th lumbar vertebra. The kidneys may also be asymmetrically located in relation to the spine. A horseshoe kidney is fixed by additional blood vessels, up to 8 for both halves, supplying the kidneys and an isthmus as well, decreasing its mobility [154]. Alongside arterial anomalies, venous anomalies may be present, with an overall incidence of 23% [155]. Considering the variability in blood supply, abdominal trauma may result in severe haemorrhage, whilst a lower location may result in renal compression over the lumbar vertebrae [134]. The anterior position of the isthmus further reduces the protective effect of the rib cage.

#### 3.6.4. Clinical Manifestation of Horseshoe Kidney Injuries

Typical clinical presentation involved pain and tenderness of the abdomen, hematuria, and hypovolemia in patients with advanced trauma.

#### 3.6.5. Diagnosis of Horseshoe Kidney Injuries

Computed tomography (CT) scan remains a gold standard for renal injury grade classification and malformation detection in stable patients [146,156,157]. Thus, the CT scan was the imaging test of choice in reviewed cases, allowing for the accurate identification of horseshoe kidneys and the extent of injuries. Focused Assessment Sonography in Trauma (FAST) is used in critical patients to identify the cause of hemodynamic instability, but it is inferior to a CT scan and does not define renal injury well. [https://uroweb.org/guidelines/urological-trauma/chapter/urogenital-trauma-guidelines] However, it was still used in three cases, followed by a CT scan to further investigate the extent of trauma [137,139,147]. In two instances, FAST was negative despite active arterial bleeding and the presence of a retroperitoneal haematoma visible during the CT scan [139,147]. MRI has the same diagnostic accuracy as CT. Nevertheless, it poses some logistic challenges limiting its use in trauma patients [157]. As stated previously, horseshoe kidneys are highly vulnerable to abdominal trauma and may prove challenging to treat; therefore, early diagnosis and management remain crucial in trauma patients [135]. The retroperitoneal location of the kidneys limits the sensitivity of FAST in detecting isolated renal injuries.

#### 3.6.6. Treatment of Injuries Associated with Horseshoe Kidney Injuries

Treatment depends on hemodynamic stability and CT scan results, with conservative treatment preferred in stable patients [146] as non-operative methods have a success rate of 94.8% [139]. Even though conservative treatment is used in most cases, it is not always possible. Minimally invasive treatment, such as embolization or stenting, is the next line of treatment; however, it may prove challenging due to the presence of vascular anomalies. Selective embolization and angiography may be used as an alternative in active bleeding cases with a high response rate of 98% [135]. If both conservative treatment and embolization are not possible due to too high risk, surgery is performed. Surgical approach consists of partial as well as total nephrectomy and nephrorrhaphy; additionally, it may be used to avoid chronic kidney failure in patients with severe injuries [135]. In this study, all patients were treated successfully, with either a conservative approach, embolization, laparotomy, stenting or drainage in case of urinoma.

#### 3.6.7. Summary of Horseshoe Kidney Injuries

In conclusion, injuries concerning horseshoe kidneys are rare but may prove difficult to treat due to their anatomy. They are mainly caused by vehicle accidents. The trauma results in haemorrhage and haematoma due to injured additional blood vessels. Additionally, a horseshoe kidney is susceptible to rupture as a result of its lower, unprotected localization. Due to the lack of definitive guidelines for horseshoe kidney trauma management, further investigation should be carried out. Compared with anatomically normal kidneys, the fused structure and aberrant vasculature increase both diagnostic and therapeutic complexity.

## 4. Discussion

### 4.1. Synthetic Overview of the Discussed Anomalies in Terms of Demographic Data and Injury Risk

The presented review reveals a noticeable discrepancy in the age of patients in the study group. All analysed anomalies, with the exception of duodenal diverticulum perforation, occurred predominantly in younger patients, although in individual series, there were cases of significantly older individuals (e.g., horseshoe kidney—a 78-year-old case; accessory spleen—rupture in a 61-year-old patient) [122,140].

Within the group of duodenal diverticular perforations, the average age was significantly higher, almost twice as old as in the other sub-groups, probably due to the predominance of pseudodiverticula, the prevalence of which increases with age [43].

The vast majority of cases have shown a predominance of males, which correlates with observations in typical abdominal injuries [158]. Reasons for this distribution can be connected with higher occupational risk, greater exposure to traffic accidents, and risky behaviour among men [158].

There is a clear dividing line here: injuries to duodenal diverticula are more common in older women, while injuries related to splenic torsion, situs inversus or horseshoe kidney are more common in young men or teenagers.

The main mechanism of injury, irrespective of the type of anomaly, is traffic accidents, in which kinetic forces, compression with accompanying increased pressure in the abdominal cavity, and the mechanism of deceleration (sudden braking) play a key role.

The highest mortality rates were observed in subgroups with injuries accompanied by situs inversus (2/7 cases; 29%) and duodenal diverticulum perforation (4/21 cases; 20%). A favourable prognosis was found in patients who had suffered blunt trauma to Meckel’s diverticulum and in patients with horseshoe kidney injuries. Early diagnosis and immediate implementation of appropriate treatment are extremely important.

A detailed comparison of demographic data and circumstances of injuries depending on anatomical anomalies is presented in Table 2.

### 4.2. Pathophysiological Elements Common to the Anomalies Analysed

Splenic torsion is most often associated with a defective ligament system—their deficiency or excessive length, while the horseshoe kidney, due to its lower position and proximity to the spine, is more vulnerable to direct injury than the physiologically located kidney [134].

Compression and local pressure increase are the primary mechanisms in blunt injuries of intestinal diverticula (duodenum and Meckel’s diverticulum). Perforation may occur either as a consequence of direct impact or as a result of sudden pressure increase in the so-called closed loop phenomenon [76].

Moreover, the pseudodiverticular wall of the duodenum is thinner than the wall of a healthy intestine, which hypothetically predisposes it to perforation with less force accompanying the injury [51]. Thus, the analysed anomalies increase injury susceptibility through three principal mechanisms: hypermobility, structural fragility, and altered anatomical positioning.

### 4.3. Impact of Anomalies on the Lack of Consistency of Symptoms with Typical Diagnostic Algorithms

In the study population, symptoms were often non-specific and could imitate more frequent traumatic injuries. In patients with situs inversus, the location of pain is reversed—pain located on the right side does not suggest liver damage, rather it corresponds to spleen damage. Such a mirror-image presentation increases the risk of misdiagnosis and diagnostic delay.

For some anatomical anomalies, some cases had delayed symptom onset. Injuries to duodenal diverticula or rupture of the accessory spleen can occur only several hours or even days after the injury. Similar delays have been observed in cases of splenic torsion as well as in some cases of situs inversus, which means that the clinical picture does not always correspond to that of a typical trauma patient requiring immediate intervention. Standard trauma algorithms are largely based on conventional anatomical positioning and predictable injury patterns; anatomical variants may therefore reduce their diagnostic reliability.

### 4.4. Key Clinical Implications in Emergency and Surgical Practice

Unspecific or delayed symptoms increase the risk of delayed surgical intervention (e.g., laparotomy in duodenal diverticular perforation), which may be associated with higher mortality.

Most of the injuries under review are associated with complications such as haematomas or retroperitoneal infections (especially in injuries to the duodenum and horseshoe kidney). Preoperative awareness of underlying anatomical anomalies is essential for appropriate surgical planning (e.g., modification of the incision in situs inversus) and for careful dissection of vessels in the horseshoe kidney, which is why meticulous interpretation of imaging studies is crucial. Failure to recognise anatomical variants preoperatively may increase the risk of intraoperative complications, including vascular injury and prolonged operative time.

### 4.5. Detailed Interpretation of Imaging Studies

Computed tomography (CT) with contrast enhancement should be considered the key diagnostic tool in all of the anomalies mentioned above. It allows for assessment of trauma and identification of unexpected anatomical variants (e.g., vascular mapping, situs inversus). Additional tests, such as ultrasound (FAST) or X-ray, serve as screening tools; however, they are less sensitive in detecting certain specific injuries (e.g., duodenal diverticulum perforation, accessory spleen injury). They should not be the sole basis for diagnosis or the final diagnosis.

In some cases, the diagnosis should be extended to include angiography, especially in horseshoe kidney injuries, where angiography allows the complexity of the vasculature to be assessed and enables embolisation to be considered as an alternative to surgical treatment.

A precise assessment of the effectiveness of imaging methods in diagnosing blunt abdominal trauma in patients with anatomical anomalies is presented in Table 3.

### 4.6. Time Stratification and the Impact of Diagnostic Methods

To reduce the impact of historical diagnostic limitations, the 91 cases analysed were divided into the ‘pre-CT era’ (before 1980) and the ‘modern CT era’ (since 1980). The vast majority, namely 82 cases (90.1%), are from the contemporary period, whereas only 9 (9.9%) are from the period prior to computed tomography.

Individual anomalies were analysed, revealing a varied distribution of historical cases. The highest proportion of patients from the pre-CT era was recorded in the subgroups of accessory spleen rupture (2 out of 9; 22.2%) and Meckel’s diverticulum (5 out of 30; 16.7%). A slightly lower proportion was observed in cases of duodenal diverticulum (2 out of 21; 9.5%). Conversely, in the subgroups of horseshoe kidney (n = 16), splenic torsion (n = 8) and situs inversus (n = 7), all the cases described occurred exclusively in the era of widespread access to modern CT.

This chronological breakdown is crucial for interpreting the treatment outcomes. It clearly shows that the highest mortality rates observed in the study—in patients with situs inversus (29%) and duodenal diverticulum (20%)—were not an artefact resulting from a lack of advanced imaging. All cases of situs inversus (7/7) and nearly all duodenal diverticulum injuries (19/21) occurred in the CT era. These findings demonstrate that diagnostic and therapeutic difficulties stem directly from the confusing clinical presentation of the anomalies themselves, rather than from historical limitations in equipment.

A detailed breakdown of cases by subcategory, both prior to the introduction of computed tomography and following its widespread adoption, is presented in Table 3.

### 4.7. The Role of Clinical Experience and a Multidisciplinary Approach

Effective diagnosis and management of rare blunt traumatic injuries require close multidisciplinary collaboration between radiologists, surgeons, anaesthesiologists, and intensive care teams. The radiologist should not only assess the presence of blood and fluid, but also actively identify anatomical variants (vascular course, reverse visceral positioning), which decreases the risk of iatrogenic mistakes during surgery.

Treatment must be tailored to each individual patient, whereas the decision between conservative and surgical management should be based on hemodynamic stability, the nature of the anomaly, and available resources (e.g., the possibility of embolization). Careful monitoring and cooperation among the surgical staff and the intensive care unit are essential for the application of appropriate treatment standards.

A summary of the therapeutic strategies used and treatment outcomes in blunt abdominal trauma in patients with anatomical anomalies is presented in Table 4.

### 4.8. Limitations of the Review

The combined group of cases analysed included 91 patients and is based predominantly on case reports and small series, which prevents comprehensive statistical analysis and the drawing of definitive conclusions. Additionally, variability in reporting quality among case reports may have influenced the completeness of extracted data.

Owing to the extensive scope of the research topic and the considerable clinical diversity of the available case reports, it was not possible to formulate a single, precise PICO question. Hence, we decided against conducting a systematic review and opted instead for a narrative review. This strategy made it possible for us to present clinical observations in a concise manner, compare case reports in terms of key diagnostic and therapeutic features, and identify recurring patterns of management that may be useful in clinical practice. Simultaneously, we deliberately adopted broad inclusion criteria to encompass rare and heterogeneous cases, which enhances the review’s utility for practitioners but simultaneously limits the possibility of conducting formal statistical analyses and formulating generalised recommendations. Consequently, the conclusions presented in this paper are descriptive and hypothesis-generating; they serve to highlight gaps in knowledge and guide future prospective studies, rather than providing direct therapeutic guidelines.

Another limitation stems from the nature and structure of our study, based primarily on case reports and small series. Naturally, this methodology introduces publication bias, strongly favouring severe, complicated injuries that often require surgical intervention. Consequently, mild cases that have been successfully treated conservatively and have a favourable prognosis are most likely overlooked or significantly underrepresented in the literature.

Furthermore, the widely defined time criteria (1960–2025) increase the heterogeneity of the data, and changes in diagnostic and therapeutic standards over the decades require caution in interpretation.

To verify the impact of historical limitations on treatment outcomes, the pre-CT era was identified, with 1979 adopted as a safe cut-off point [159,160,161]. It should be noted that only a small proportion of the analysed cases originate from the pre-CT era. What is extremely significant from a clinical perspective is that the temporal stratification revealed that the highest observed mortality rate—contrary to initial assumptions—is not merely a historical artefact resulting from the lack of advanced imaging. In the subgroups with the worst prognosis, such as situs inversus (mortality 29%) or perforation of a duodenal diverticulum (mortality 20%), all (7 out of 7) or the vast majority (19 out of 21) of the cases described occurred in the ‘modern CT’ era. This demonstrates that treatment difficulties stem from the specific nature of the anomalies themselves—a misleading clinical picture (such as mirrored pain localisation in situs inversus) or the delayed onset of non-specific symptoms in older patients (duodenal diverticula), which leads to diagnostic errors even with access to computed tomography. Nevertheless, it should be remembered that an objective limitation of our review remains the inability to determine precisely when individual centres around the world actually gained access to this imaging technology.

Despite these limitations, our observations and conclusions are formulated in a very cautious and conservative manner. Our review provides the most comprehensive and rigorous summary of available case reports concerning blunt trauma in the context of rare anatomical anomalies. It serves as a beneficial resource for clinicians and researchers, highlighting gaps in knowledge and areas requiring further prospective investigation.

## 5. Conclusions

In the context of blunt abdominal trauma associated with rare anatomical anomalies, contrast-enhanced computed tomography (CECT) serves as the pivotal diagnostic tool, enabling not only injury assessment but also precise mapping of aberrant anatomy, which is crucial for subsequent clinical management. The highest mortality rate was recorded in injuries accompanied by situs inversus (2/7) and duodenal diverticulum perforation (4/21). Crucially, temporal stratification demonstrated that these fatalities occurred predominantly in the modern CT era, indicating that poor outcomes are driven by the misleading clinical picture and delayed diagnosis rather than historical limitations in imaging. Compression and increased pressure play a significant role in the pathomechanism of duodenal and Meckel’s diverticulum injuries, while deceleration forces dominate in horseshoe kidney and spleen torsion injuries. Although rare anatomical anomalies often require urgent surgical intervention, modern imaging techniques (CECT) allow for the effective use of conservative management in haemodynamically stable patients; this is particularly evident in cases of horseshoe kidney, which represent a significant departure from the traditional surgical paradigm. Duodenal diverticulum perforations predominantly affect older women (average age 62), while injuries in the course of other anatomical anomalies are characteristic of young men. Awareness of rare anatomical variants and their impact on trauma patterns is essential for reducing diagnostic delay and improving patient outcomes in emergency and surgical practice.

## Figures and Tables

**Table 1:** Literature search strategy and eligibility criteria applied in the review.

Variable		Details
**Searches of Databases**		PubMed, Scopus, Web of Science, Google Scholar
**Time Frame**		1960–2025 (last update: December 2025)
**Search Strategy**		Two mandatory conceptual blocks combined using Boolean operators (AND/OR); MeSH terms and free-text keywords
**Terms Related to**	**Trauma**	Blunt abdominal trauma, blunt abdominal injury, parenchymal organ injury
	**Anatomical Anomaly**	Duodenal diverticulum, Meckel’s diverticulum, situs inversus, horseshoe kidney, splenic torsion, wandering spleen, accessory spleen
**Inclusion Criteria**		- Patients of any age; - confirmed blunt abdominal trauma with tissue damage (e.g., perforation, rupture, haemorrhage, ischaemia); - pre-existing anatomical anomaly confirmed by imaging or intraoperatively; - case reports, case series, and original scientific articles
**Exclusion Criteria**		- Non-blunt or unclear injury mechanism (e.g., penetrating, iatrogenic); - incidental anomalies without traumatic relevance; - animal studies; - grey literature (conference abstracts, unpublished data)

**Table 2:** Demographic characteristics of patients with rare anatomical anomalies following blunt abdominal trauma, including the mechanisms of injury.

Anatomical Anomalies Injuries (n)	Mean Age (Range)	Gender		M:F Ratio	Frequent Mechanism of Trauma
		Males n (%)	Females n (%)		
Duodenal diverticulum (n = 21)	62 (±19)	9 (43%)	12 (57%)	0.75	Traffic accidents (43%), falls from height (38%)
Meckel’s diverticulum (n = 30)	27.1 6–60	29 (96.7%)	1 (3.3%)	27	Traffic accidents, followed by sports accidents, accidents at work, and assaults
Splenic torsion (n = 8)	16.3 (5–28)	4 (50%)	4 (50%)	1	Traffic accidents, trauma predisposing to subsequent secondary torsion
Accessory spleen rupture (n = 9)	35 (8–61)	8 (89%)	1 (11%)	8	Direct blunt abdominal trauma
Situs inversus (n = 7)	23 (13–30)	5 (71%)	2 (29%)	2.5	Traffic accidents (43%)
Horseshoe kidney (n = 16)	32.1 (10–78)	14 (87.5%)	2 (12.5%)	7	Traffic accidents (50%), sudden deceleration

**Table 3:** Effectiveness of imaging methods in diagnosing blunt abdominal trauma in patients with anatomical anomalies.

Anatomical Anomalies Injuries (n)	Pre-CT Era Cases (n) *	Modern CT Era Cases (n) ^x^	CT	USG/FAST	X-Ray
Duodenal diverticulum (n = 21)	2	19	Very High	60%	33%
Meckel’s diverticulum (n = 30)	5	25	High	Moderate	Moderate
Splenic torsion (n = 8)	0	8	High	Appropriate (Doppler)	N/A
Accessory spleen rupturę (n = 9)	2	7	Low (interference: size, haemorrhage)	weak	N/A
Situs inversus (n = 7)	0	7	High (diagnostics + anatomical map)	Appropriate (screening)	Good (confirmation)
Horseshoe kidney (n = 16)	0	16	Very High	weak (false negative)	N/A

*—Before 1980 (the era previous to the widespread implementation of CT), ^x^—Since 1980 (a time when CT scans may already have been in widespread use). N/A—not applicable

**Table 4:** Therapeutic strategies and treatment outcomes for blunt abdominal injuries in the course of anatomical anomalies.

Anatomical Anomalies Injuries	Predominant Treatment	Surgical Treatment	Main Procedure	Prognosis/Mortality
**Duodenal diverticulum**	Surgical	95% (20/21)	Diverticulectomy, Drainage	20% (4/21)
**Meckel’s diverticulum**	Surgical	100% (30/30)	Diverticulectomy, enterectomy	Full recovery
**Splenic torsion**	Surgical	100% (8/8)	Splenectomy (rarely splenopexy)	Risk of OPSI, organ loss
**Accessory spleen rupture**	Surgical	78% (7/9)	Laparotomy	Beneficial in intervention; compensatory hypertrophy possible
**Situs inversus**	Surgical	86% (6/7)	Splenectomy, enterectomy, hemihepatectomy	29% (2/7)
**Horseshoe kidney**	Conservative	25% (4/16)	Observation, embolisation, stenting	No deaths; 94.8% effectiveness

## Data Availability

No new data were created or analyzed in this study.

## Abbreviations

The following abbreviations are used in this manuscript:

AS	Accessory Spleen
CECT	CECT
D2	Descending Duodenum
D3	Horizontal Duodenum
DD	Duodenal Diverticulum
ERCP	Endoscopic Retrograde Choleangipancreatography
F	Females
FAST	Focused Assessment with Sonography for Trauma
M	Males
MD	Meckel’s Diverticulum
MeSH	Medical Subject Headings
MRI	Magnetic Resonance Imaging
n	Number (sample size)
N/A	Not Applicable/Not Available
OPSI	Overwhelming Post-Splenectomy Infection
SD	Standard Deviation
USG	Ultrasonography
AS	Accessory Spleen
CECT	CECT
D2	Descending Duodenum
D3	Horizontal Duodenum
